# Trajectories of Childbearing among HIV Infected Indian Women: A Sequence Analysis Approach

**DOI:** 10.1371/journal.pone.0124537

**Published:** 2015-04-23

**Authors:** Shrinivas Darak, Melinda Mills, Vinay Kulkarni, Sanjeevani Kulkarni, Inge Hutter, Fanny Janssen

**Affiliations:** 1 Population Research Centre, Faculty of Spatial Sciences, University of Groningen, Groningen, The Netherlands; 2 PRAYAS Health Group, Pune, Maharashtra, India; 3 Department of Sociology, Nuffield College, University of Oxford, Oxford, England; 4 Netherlands Interdisciplinary Demographic Institute, The Hague, The Netherlands; UCL Institute of Child Health, University College London, UNITED KINGDOM

## Abstract

**Background:**

HIV infection closely relates to and deeply affects the reproductive career of those infected. However, little is known about the reproductive career trajectories, specifically the interaction of the timing of HIV diagnosis with the timing and sequencing of reproductive events among HIV infected women. This is the first study to describe and typify this interaction.

**Methods:**

Retrospective calendar data of ever married HIV infected women aged 15-45 attending a HIV clinic in Pune, Maharashtra, Western India (N=622) on reproductive events such as marriage, cohabitation with the partner, use of contraception, pregnancy, childbirth and HIV diagnosis were analyzed using sequence analysis and multinomial logistic regression.

**Results:**

Optimal matching revealed three distinct trajectories: 1) HIV diagnosis concurrent with childbearing (40.7%), 2) HIV diagnosis after childbearing (32.1%), and 3) HIV diagnosis after husband’s death (27.2%). Multinomial logistic regression (trajectory 1 = baseline) showed that women who got married before the age of 21 years and who had no or primary level education had a significantly higher risk of knowing their HIV status either after childbearing or close to their husband’s death. The risk of HIV diagnosis after husband’s death was also higher among rural women and those who were diagnosed before 2005.

**Conclusions:**

Three distinct patterns of interaction of timing of HIV diagnosis with timing and sequencing of events in the reproductive career were observed that have clear implications for (i) understanding of the individual life planning process in the context of HIV, (ii) formulation of assumptions for estimating HIV infected women in need of PMTCT services, and (iii) provision of care services.

## Introduction

Among the most significant achievements of the global response to the AIDS epidemic in the last decade is the remarkable scale up of Antiretroviral Treatment (ART) and the rapid expansion of services for Prevention of Mother To Child Transmission (PMTCT) of HIV in developing countries. Globally, 35 million people are estimated to be living with HIV by the end of 2013 [1]. ART reached 12.9 million HIV infected people by the end of 2013, of which 5.6 million people were added in just three years since 2010 [1].There is a global plan towards elimination of new HIV infections among children by 2015 and keeping mothers and children living with HIV alive [2]. Many low-and middle-income countries have already moved significantly towards achieving these goals. In 2013, twice as many (68%) pregnant women living with HIV in the countries with high HIV burden had access to antiretroviral medicines to reduce the risk of HIV transmission to their children compared to 2009 (33%) [3]. This rapid expansion of ART to infected people and reaching out to HIV infected pregnant women with PMTCT has instigated hope of ending the AIDS epidemic in the world. Recently, new global targets for the year 2020 have been launched at the United Nations General Assembly. The aim is, by 2020, 90% of all people living with HIV will know about their HIV status, 90% of all people diagnosed with HIV will receive treatment and 90% of people on treatment will achieve viral suppression [4].

India, which has a HIV prevalence of 0.27% [5] and by the end of 2012 has an estimated 2.08 million HIV infected inhabitants aged 15–49 [5], is the third largest country in terms of the number of people living with HIV (PLHIV).

With the significant expansion of ART and PMTCT services, the health status of people living with HIV/AIDS is improving significantly. In India, HIV testing, particularly for pregnant women through integrated counseling and testing centers (ICTCs), was started in 2002 in a few centers. There has been a remarkable increase in the coverage of HIV testing, ART and PMTCT services in last few years. It has now expanded across the nation and provides counseling and testing services through more than 15,000 ICTCs [5]. The 2013–2014 annual report of the Department of AIDS control, previously called the National AIDS Control Organization of India (NACO), reports that more than 13 million people (general population excluding pregnant women) and 9.7 million pregnant women (74% of the total pregnant women in the country) were tested for HIV. The free ART program which started in the year 2004 in 8 government hospitals has been expanded to more than 400 centers in the country [5] and is currently providing treatment to more than 54% of the HIV infected people [6]. The morbidity and mortality associated with AIDS and rates of mother to child transmission have reduced. It is estimated that in the last decade the incidence of HIV infection in India has also decreased by more than 50 percent [7]. The ensuing hope of a healthy and long life warrants attention towards issues related to quality of life of PLHIV including their reproductive careers.

HIV infection closely relates to and deeply affects the reproductive health and reproductive choices of those infected [8]. The goal of achieving the status of mother is central in the life of most Indian women. However, in the context of HIV infection, the path of achieving motherhood is fraught with dilemmas and challenges. As any sexually transmitted infection associated with childbearing and breastfeeding [9,10], knowledge of one’s HIV status can significantly affect reproductive decision-making among couples when one or both the partners are HIV infected. Women, who choose to conceive or continue their pregnancy after being detected as HIV infected, have to cope with the fear of transmitting the virus to the child and subsequent guilt if the child is infected [11]. Those who want to avoid this and decide not to conceive or to terminate pregnancy, have to bear its negative social consequences. On the other hand, having a child can often be the only solace in the otherwise disrupted life of a HIV infected woman. Motherhood therefore can intensify or mitigate the negative consequences of HIV infection [12,13].

With the increasing expansion of PMTCT program in India, it can be expected that the timing of HIV diagnosis with respect to the timing of events in the reproductive career of women would also change. An increasing proportion of women are likely to become aware about their HIV positive status during their first pregnancy. Though with ARVs the possibility of HIV transmission to the baby can be reduced to less than 2% compared to 25–30% without any intervention [10], knowledge about HIV status still can have significant effect on the reproductive decision-making of the couples.

There is paucity of quantitative research adopting a life course perspective examining events in the reproductive career of HIV infected women. Previous research about reproductive issues among HIV infected women has been restricted to understanding fertility desires among HIV infected women [14], the complexities in reproductive decision-making [15], assessing the impact of HIV on reduction of fertility [16] and documenting adverse pregnancy outcomes [17,18]. There is a recent emphasis on studying the impact of ART on fertility desires and pregnancy outcomes among HIV infected women [19,20] and the role of preconception counseling in reducing unwanted pregnancies among HIV infected couples [21]. As conception among HIV infected couples with concordant or discordant HIV status is associated with some risk of HIV transmission to the partner and/or baby, making conception safer among HIV infected couples has remained at the core of the research regarding reproductive health issues among HIV infected couples. The different reproductive events among HIV infected women have mostly been studied independently. What remains unknown is how the trajectories of events in the reproductive career of HIV infected women influence the interaction of the timing of HIV diagnosis with different reproductive events. Such an analysis of two trajectories over a period of time (diachronic interaction), i.e. the analysis of events regarding HIV diagnosis of the woman and her partner over a period of time; and the events in the reproductive career (pregnancies, childbirths, marriage dissolution) of the woman occurring over that period of time, would help us to understand the individual life planning underpinning the timing of reproductive events. It would also provide insights for estimating the number of HIV infected women in need of PMTCT services.

The objective of this paper is to describe and typify the trajectories of events in the reproductive career of HIV infected women in Western India taking into account the interaction of the timing of HIV diagnosis with different reproductive events.

We used the life course perspective to analyze the trajectories of events in the reproductive career of HIV infected women [22]; wherein the reproductive career of women starts with the event of menarche and is followed by life events such as marriage (or more relevantly the beginning of sexual relationship), pregnancies, outcomes of those pregnancies (live births, still births, spontaneous abortions, induced abortions) and ends with menopause or sterilization [23]. In the Indian context and especially with reference to HIV, marriage can be considered as a start of the reproductive career as almost all women report to begin their sexual activity only with marriage and thus acquire HIV infection after marriage through their husbands [24].

Factors associated with HIV infection among young Indian women are probably unique compared to most other countries with higher rates of HIV transmission. Most Indian women acquire HIV infection from their husbands at a very young age [24]. The median age at marriage among Indian women is 17.2 years and the median age at first birth is 20 years [25]. Use of temporary contraceptive methods (mainly male condoms) for child spacing is very low with only 6% of couples using contraception to space pregnancy, [25] suggesting high social pressure on women to prove their fertility soon after marriage. Though the fertility rates in India are declining with current total national fertility rate of 2.7 [25], there is wide variation in fertility rates across different states in India. It is important to note that the Southern states- Andhra Pradesh, Karnataka, Maharashtra and Tamil Nadu, which have the highest burden of HIV in India have fertility levels at or below replacement levels [25]. These states also have very high rates of female sterilizations compared to rest of the India. While female sterilization is by far the most preferred method of contraception across India with 37% of the currently married women having been sterilized nationally, 51–63% of the currently married women were sterilized in these 4 states [25]. Importantly, more than half of these women get sterilization operations before they reach 26 years of age [25]. This leads to a very short time span from marriage to the occurrence of sterility (effective reproductive span) [26].

Considering this specific context, understanding the reproductive career trajectories of HIV infected Indian women would have significant implications for the PMTCT programs in the country.

## Materials and Methods

### Study setting

The study was conducted among HIV infected women attending a specialty HIV clinic run by a non-government organization (NGO) in Pune, Maharashtra, Western India. Maharashtra has a high HIV prevalence (for 2013: 0.40) [5].

### Study population

From November 2010 until December 2011 all HIV infected women attending the clinic were screened for eligibility (N = 1,023). Ever married women aged 15–45 and who knew about their HIV positive status for more than 6 months were considered eligible and were informed about the study. Of the eligible women (N = 820), 99% (N = 811) were informed about the study and 622 (77%) participated and completed the interview.

### Ethical considerations

The study protocol, consent forms and data collection tools were reviewed and approved by the organization’s Independent Ethics Committee for Research (IECR). The ethics committee is registered with the government agency (registration number: ECR/146/Indt/MH/2014). Informed written consent was obtained from the women prior to data collection and confidentiality norms were strictly adhered to. Counseling support was available to women when required.

### Data collection

We collected retrospective calendar data on all the reproductive events starting from menarche to menopause. Information about the timing (month and year) of the events pertaining to the domains of marriage (married, divorced and widowed) and cohabitation with partner (yes/no), childbearing (pregnancies, births and other pregnancy outcomes), contraceptive use and treatment seeking was collected by trained woman interviewers using the conversational interviewing approach. Information about other demographic variables was collected from the women at the time of interview which included education, place of living (rural/urban), socio-economic status (Kuppuswamy scale [27]), dates of HIV testing of the woman and her partner and reason for HIV testing. Partner’s HIV status was recorded as reported by women.

### Data preparation

The data on dates of different events were arranged on a time scale corresponding to the age of the women starting from 10 years until 40 years. In cases when the women’s age was less than 40 years, the observation was censored by the interview date. Each column corresponded to a specific time unit (months) and included data on a mutually exclusive state (See below).

#### Different states

We selected 15 different states to analyze the interaction of HIV diagnosis with reproductive events. The occurrence and timing of the reproductive events in the two distinct domains of marriage and childbearing and its interaction with the third domain of HIV diagnosis was analyzed.

Marital status was dichotomized into: 1) currently married or 2) not currently married. Childbearing was considered as 1) parity zero 2) parity one and 3) parity two and over, where parity indicates the number of children the woman has already given birth to. The retrospectively constructed trajectory of HIV diagnosis based on the dates of HIV testing of the woman and her partner was categorized into four categories based on HIV testing and the consequent knowledge of the HIV status of the woman and her partner. These are:1) both husband and wife are not tested for HIV and hence not aware about their HIV status, 2) only one of the couple (either husband or wife) was tested for HIV and is HIV infected however the other partner did not test for HIV and hence his/her HIV status is not known, 3) both husband and wife are HIV infected and aware of it (concordant), 4) the wife is HIV infected and the husband is not infected and both are aware of their HIV status (discordant). See Table 1 for a description of the 15 different states used in the analysis.

**Table 1:** Description of states used in the analysis.

HIV diagnosis states	Code	Marital status	Awareness of HIV status	Childbearing
Neither she nor her husband is tested for HIV and hence are unaware (NA) of their HIV status	(M-NA-P0)	Married	Not Aware	Parity 0
	(M-NA-P1)	Married	Not Aware	Parity 1
	(M-NA-P2+)	Married	Not Aware	Parity 2+
One of the couple (A1) (either she or her husband) is tested HIV positive and aware about his/her HIV status.	(M-A1-P0)	Married	One partner positive	Parity 0
	(M-A1-P1)	Married	One partner positive	Parity 1
	(M-A1-P2+)	Married	One partner positive	Parity 2+
Both she and her husband are tested HIV positive and are aware about it-concordant (C)	(M-C-P0)	Married	Concordant	Parity 0
	(M-C-P1)	Married	Concordant	Parity 1
	(M-C-P2+)	Married	Concordant	Parity 2+
She is tested HIV positive and her husband is tested HIV negative and both are aware about their respective HIV statuses- Discordant (D)	(M-D-P0)	Married	Discordant	Parity 0
	(M-D-P0)	Married	Discordant	Parity 1
	(M-D-P0)	Married	Discordant	Parity 2+
She is not tested for HIV and hence not aware (NA) about her HIV status	(W-NA)	Widow	Not Aware	Not considered
She is tested HIV positive and aware about it	(W-A)	Widow	Aware	Not considered
	U	Unmarried		Not applicable

### Data analysis

Descriptive statistics such as median and inter-quartile range was provided for skewed continuous data such as age and duration of reproductive span. To understand the timing and sequencing of reproductive events and its interaction with HIV diagnosis we used sequence analysis which is the analysis of categorical sequences of events to model different events in the trajectories taking into account the order in which events occurred and the transition mechanism between different states [28].

#### Analytical approach

The data analysis included three steps: 1) description of the timing of reproductive events such as marriage, marriage dissolution, pregnancy, childbirth and sterilization 2) deriving typologies of the reproductive career and 3) analyzing the association of socio-demographic variables with the derived typologies.

We describe the timing of events in the reproductive life of HIV infected women who experienced the events by estimating the median ages of the women at which these events occurred.

The typologies of reproductive career trajectories were derived by clustering the common sequences from the data by using optimal matching (OM). Optimal matching (OM) is a well-established technique for comparison of sequences [29]. In OM, the distance between sequences is quantified as the minimum number of edits required to generate identical sequences [30] such as insertion, deletion or substitution. Two sequences are considered similar based on the number of features they share in common. We plotted all the sequences in a graph (dendrogram) to examining the clustering of the sequences [31]. For more robust analysis we opted to choose three clusters of the sequences as 4 or 5 clusters led to too few cases in a few clusters.

The typical reproductive career trajectories within each cluster are described by estimating the most representative (central) sequences using *medoid sequences* and by estimating sequences that represent 25% of the data in each cluster [32,33]. The medoid sequence is the individual sequence that is least distant from all other sequences in the data [34]. The mediod individual is thus an actual person. With this approach, it is possible to describe a cluster by its mediod (representative person in the cluster).

The association of socio-demographic factors with these derived typologies is estimated in a multinomial logistic regression model, with most commonly occurring typology as a reference category. While building the multinomial model, the socio-demographic factors (Table 2) such as education, place of residence and socio-economic status that are known to affect health care utilization among women [35] were considered as independent variables along with the variable of year of HIV diagnosis for assessing the possible change after the beginning of ART and PMTCT programs in Maharashtra (Table 2). Variables were tested using main effects model. Statistical significance was determined by examining the confidence intervals of the relative risk ratios and the corresponding p-values of the coefficients. A p-value of less than 0.05 was considered as statistically significant. Variables with statistically significant values were described in Table 3 and Table 4.

**Table 2:** Profile of HIV infected women in the study (N = 622).

	Variable	Category	N	%
Demographic factors	Age at marriage	< = 17 year	277	44.5
		18–21 years	230	37.0
		> = 22 years	115	18.5
	Education	No schooling or Primary	216	34.7
		Secondary or Higher	406	65.3
	Socio-economic class	Upper	339	54.5
		Lower	283	45.5
	Place of residence	Urban	364	58.5
		Rural	258	41.5
	Age difference with husband (husband older than woman)	0–4 years	156	25.1
		5–8 years	249	40.0
		= < 9 years	217	34.9
Marital factors	Marital status at interview	Living as married	366	58.8
		Divorced	6	1.0
		Widowed	235	37.8
		Separated	15	2.4
	Ever had live birth	Yes	548	88.1
		No	74	11.9
	Ever use of contraception	Female sterilization	239	38.4
		Condom	449	72.2
		Pill	112	18.0
		IUD	116	18.6
		Emergency pills	15	2.4
HIV related factors	Reason of women’s HIV testing	During ANC	190	30.5
		Husband tested positive	212	34.1
		Illness	147	23.6
		Other	73	11.7
	Age of women’s at testing	<19	66	10.6
		20–24	192	30.9
		25–29	192	30.9
		>30	172	27.7
	Husband’s HIV status	Infected	494	79.4
		Un-infected	48	7.7
		Not tested/don’t know	80	12.9
	Year of HIV diagnosis (women)	Before 2000	117	18.8
		2001–2005	262	42.1
		2006–2011	243	39.1

**Table 3:** Description of sample of women in each identified cluster (Total N = 622) with respect to variables in multinomial regression model.

	HIV diagnosis concurrent with childbearing		HIV diagnosis after childbearing		HIV diagnosis after husband’s death		Total	
Variables	N	%	N	%	N	%	N	%
**Age at marriage**								
< = 17 years	65	26	119	59	93	55	277	45
18–21 years	101	40	72	36	57	34	230	37
> = 22 years	87	34	9	5	19	11	115	18
**Women’s Education**								
No education or primary school	*49*	*19*	*90*	45	77	46	216	35
Secondary or higher school	204	81	110	55	92	54	406	65
**Place of residence**								
Urban	168	66	110	55	86	51	364	58.5
Rural	85	34	90	45	83	49	258	41.5
**Year of HIV diagnosis**								
Before 2000	37	15	27	13	53	31	117	19
2001–2005	107	42	77	39	78	46	262	42
2006–2011	109	43	96	48	38	23	243	39
**Total**	**253**	**41**	**200**	**32**	**169**	**27**	**622**	**100**

**Table 4:** Relative risk according to statistically significant demographic variables of belonging into one of the identified clusters (reference group: HIV diagnosis concurrent with childbearing).

	HIV diagnosis after childbearing		HIV diagnosis after husband’s death	
Variables	*RR*	95% CI	*RR*	95% CI
**Age at marriage**				
< = 17 years	13.12**	(5.93–29.05)	3.82**	(1.94–7.51)
18–21 years	6.35**	(2.99–13.50)	2.20*	(1.19–4.08)
> = 22 years (Ref)	1	-	-	-
**Women’s Education**				
No education or primary school	1.67*	(1.03–2.71)	2.42**	(1.43–4.08)
Secondary or higher school (Ref)	1	-	-	-
**Place of residence**				
Urban	0.86	(0.57–1.31)	0.63*	(0.41–0.98)
Rural (Ref)	1	-	-	-
**Year of HIV diagnosis**				
Before 2000	0.97	(0.53–1.79)	5.21**	(2.85–9.53)
2001–2005	0.84	(0.54–1.30)	2.20**	(1.33–3.63)
2006–2011 (Ref)	1	-	-	-

Notes: Cox and Snell R^2^ = 0.222;

*p<0.05;

** p<0.01;

RR = Risk Ratios.

The sequence analysis was performed in the statistical software R using the TraMineR library [31] whereas the multinomial regression was conducted in SPSS (version 20).

## Results

### Timing of reproductive events

Among 622 ever married HIV infected women the median age (IQR) at first marriage is 18 (16, 20) years, which—in the Indian context—can be considered the start of sexual activity. Marriage dissolution (N = 256; 41.2%) was mostly due to widowhood (91.8%), which occurred at a median age of 27.4 (23.3, 30.8) years. Six women were divorced and 15 were separated from their husband at the time of interview.

Time from marriage to sterilization (effective reproductive span indicating the biological ability of women to reproduce) and from marriage to widowhood (social reproductive span indicating end of reproduction due to dissolution of partnership), [26] was very short. Among the women who underwent sterilization irrespective of their marital status at the time of interview (N = 270), the median duration of effective reproductive span was 6 (5, 8) years. Among the women who at the time of interview were widowed and not sterilized (N = 167), the median duration of social reproductive span was 11.4 (5.6, 13.3) years.

The median age of knowing about their own HIV positive status was 26 (22, 30) years. Twenty eight percent women came to know about their HIV status after they had completed their family size. Of the 232 women who opted sterilization for family planning, the median age was 23.5 (21.0, 26.0) years. In the total sample of 622 women, 20% had at least one pregnancy after knowing about their HIV positive status whereas 37% of the women had completed their reproductive career before knowing about their HIV positive status The median age at interview was 33 (29, 37) years. Women were tested for HIV either because their partners were tested positive (34.1%) or during antenatal visit (30.5%) or due to their own illness (23.6%). (Table 2)

At the time of interview, 58.8% women were living as married and 37.8% women were widowed. The majority of the women reported that their partner was HIV infected (79.4%) whereas 7.7% reported that their partners were uninfected and 12.9% reported that their partners were not tested for HIV. On an average, partners were 7.2 years older than the women.

### Typologies of reproductive career trajectories

Based on the sequencing of reproductive events and its interaction with the timing of HIV events, three clusters of trajectories emerged from the data.

#### 1) HIV diagnosis concurrent with childbearing (N = 253)

The women in this cluster were tested for HIV early in their reproductive career and came to know about their HIV positive status mostly during their first pregnancy (P0) (Fig 1.1). For these women knowledge about their HIV positive status and childbearing thus occurred simultaneously. The discordance (D) among couples was only observed in this cluster, suggesting that this cluster exhibits a group of women who acquired HIV infection from another source than their husbands.

**Fig 1:**
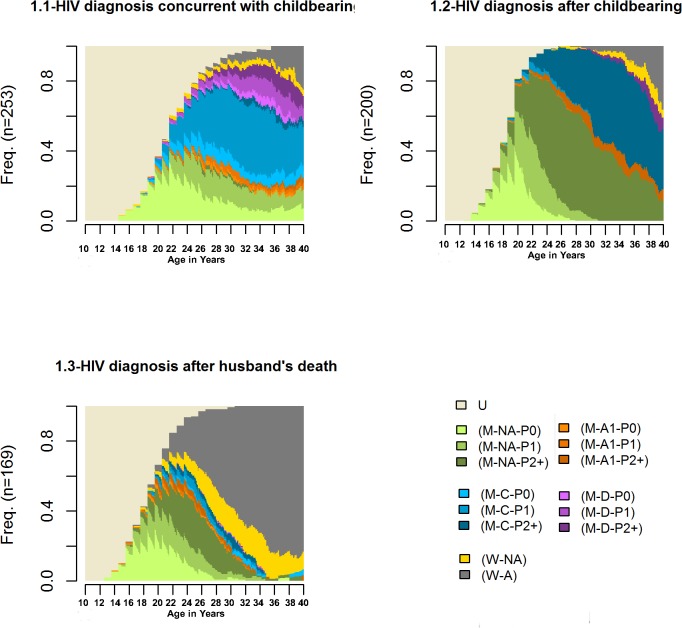
Clustered typologies of reproductive career trajectories of ever married HIV infected Indian women (N = 622). **U**- unmarried; **M**-married; **W**-widowed; **NA**-both the woman and her husband are not aware of their HIV status; **A1**–only one partner is aware of his/her HIV status; **C**-concordant (the woman and her husband are known HIV infected); **D**-discordant (only woman is known HIV infected and husband is HIV uninfected); **P0**- parity zero; **P1**-parity one; **P2+**-parity two and over. See Table 1 for a detailed description of the different states.

The most central or typical trajectory was *getting married at the average age of 21*.*5 years→ pregnant within a year of marriage→ getting tested HIV positive during the first pregnancy→ partner getting tested HIV positive immediately thereafter→ living concordant with one child*.

Four representative sequences (medoids covering at least 25% of the data in the cluster) were identified which covered 28.9% of the data (Fig 2.1). Except for the trajectory of women who were discordant (second trajectory from the top) the other three trajectories were more or less similar in terms of events in the career but differed only in terms of duration of these events. The sequencing of events in these trajectories was similar to the sequence represented in the typical trajectory of women in this cluster. The pattern in this cluster also suggested that when the woman or her partner became aware of their HIV positive status at parity 0 then they were more likely to stay childless.

**Fig 2:**
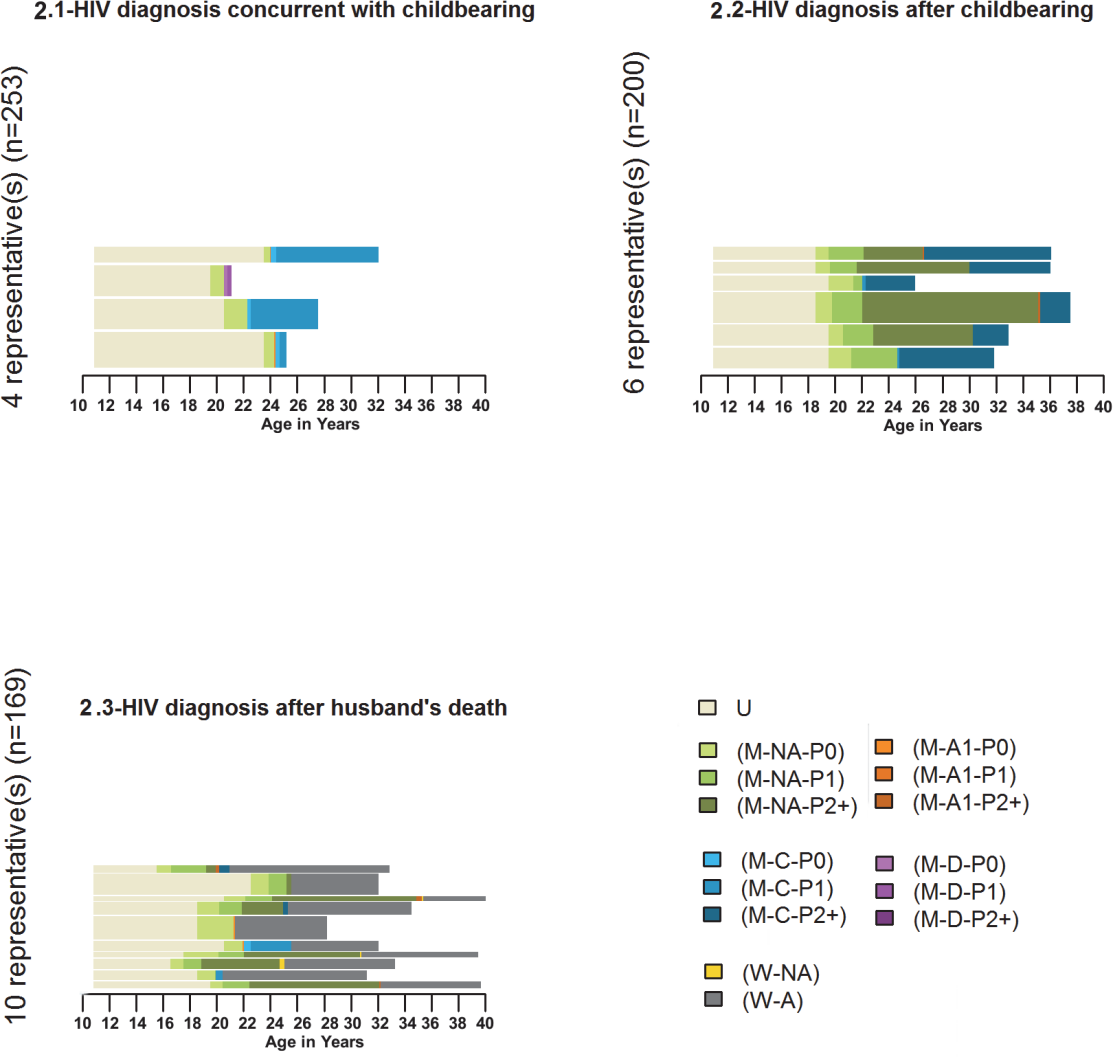
Representative sequences within each identified cluster. **U**- unmarried; **M**-married; **W**-widowed; **NA**-both the woman and her husband are not aware of their HIV status; **A1**–only one partner is aware of his/her HIV status; **C**-concordant (the woman and her husband are known HIV infected); **D**-discordant (only woman is known HIV infected and husband is HIV uninfected); **P0**- parity zero; **P1**-parity one; **P2+**-parity two and over. See Table 1 for a detailed description of the different states.

#### 2) HIV diagnosis after childbearing (N = 200)

Women in this cluster became aware of their HIV positive status after they had two or more children (P2+). Women in this cluster were either diagnosed late or acquired HIV infection at a later stage with respect to their trajectory of childbearing. (Fig 1.2)

The typical trajectory was *getting married at the average age of 22*.*9 years→1* ^*st*^ *child after 1*.*5 years→ 2* ^*nd*^ *child 2 years after the first → 3* ^*rd*^ *child 3*.*5 years after the second → one partner tested HIV positive (mostly the husband) → second partner tested HIV positive (mostly the woman) in a month → living concordant*.

Six mediod sequence clusters were identified which covered 26% of the data (Fig 2.2). Among the 1^st^, 2^nd^, 4^th^ and 5^th^ representative sequences, the woman (or her partner) became aware of her/his HIV positive status after the woman had given birth to two (or more) children and in the remaining two sequences, it was during second pregnancy of the woman. The second partner was tested HIV positive soon after the diagnosis of the first partner and the couple was living as married.

#### 3) HIV diagnosis after husband’s death (N = 169)

Widowhood (W) was a dominant feature for women in this cluster (Fig 1.3). It appeared that women became aware of their HIV positive status at a similar time when they lost their husband.

The typical trajectory was *getting married at the average age of 18*.*5 years→ for 2*.*9 years both partners were unaware of their HIV status and the woman had not given birth to any child →one partner (husband) is tested HIV positive→ woman was tested HIV positive in a month of partner’s test and knows about it and was widowed*.

This cluster showed considerably more heterogeneity compared to the other two clusters. Ten medoids were identified that could cover 26% of the data in this cluster (Fig 2.3). The most prominent trajectory (5^th^ from the top) included women who were widowed before having any child. In most other trajectories, widowhood occurred after the women had given birth to the second child.

### Socio-demographic characteristics associated with the typologies of reproductive career

The socio demographic factors of the women in the study are shown in Table 2 whereas Table 3 shows the characteristics of women in the three clusters with respect to variables that were statistically significant in the multinomial model. The relative risk of knowing about HIV status after childbearing was statistically significantly higher among women who got married before the age of 21 years (< = 17 years: RR = 13.12, 95% CI = 5.93–29.05; 18–21 years: RR = 6.35, 95% CI = 2.99–13.50) and who had no or primary level education (RR = 1.67, 95% CI = 1.03–2.71) (Table 4). The relative risk of knowing about HIV status close to the time of death of the husband (widowhood) was higher among those married at an earlier age (< = 17 years: RR = 3.82, 95% CI = 1.94–7.51; 18–21 years: RR = 2.20, 95% CI = 1.19–4.08), had no or primary level education (RR = 2.42, 95% CI = 1.43–4.08) and tested for HIV before 2005 (< = 2000: RR = 5.21, 95% CI = 2.85–9.53; 2001–2005: RR = 2.20, 95% CI = 1.33–3.63). Women who resided in an urban area were significantly more likely to know about their HIV status at the time of childbearing compared to knowing about their HIV status at the time of husband’s death (RR = 0.63, 95% CI = 0.41–0.98) (Table 4).

## Discussion

To our knowledge this is the first study to formally analyze the timing and sequencing of events in the reproductive career of HIV infected women and derive typologies of their reproductive career considering the interaction of reproductive events with timing of HIV diagnosis. Our analysis, which involved the sequence analysis technique, made it possible to identify the three prominent trajectories of the reproductive career of HIV infected Indian women and the associated factors with these trajectories. This identification is helpful for an understanding of the individual life planning processes and for guiding care, prevention and counseling, as well as for aiding the estimation of HIV infected women in need of PMTCT services.

### Implications

#### The three prominent trajectories

From the perspective of HIV prevention (to the partner as well as to the baby), the cluster of women who became aware of their HIV positive status approximately at the time of childbearing (Cluster: HIV diagnosis concurrent with childbearing) seems to have an advantage as care and support services can be provided to these women at appropriate time due to early detection. It also appears that an observed pattern of HIV discordance (wife HIV infected and husband uninfected) can be identified only when HIV diagnosis appears soon after marriage and mostly during the first pregnancy. Identification of HIV sero-discordance within couple is most likely when HIV testing is performed early. These findings highlight the importance of HIV testing of men in the PMTCT programs or possibly even earlier than first pregnancy for preventing HIV transmission within couples. This study included only HIV infected women and hence an important group of discordant couples where the husband is HIV infected and the wife is uninfected with HIV were excluded. In the Indian context where majority of HIV transmissions to women occurs within marriage, understanding the timing of events in this discordant group (husband infected and wife uninfected) would be important for prevention of HIV transmission among women.

In the cluster of HIV diagnosis concurrent with childbearing, progression to higher parity was less frequently observed when either of the partners was diagnosed before having any child. This indicates that there might be a significant decline in fertility after knowing about HIV. Desire to have children and the subsequent risk of HIV transmission due to unprotected sex among discordant couples have been documented in studies in Africa [36,37]. However our results of lower fertility among couples after knowing about their HIV status suggests that HIV infected couples might be somewhat better in dealing with external pressures regarding childbearing as they appear to succeed in restricting their fertility after knowing about their HIV status. However, the socio-cultural determinants of fertility could be significantly different among Indian women compared to African women demanding context specific in-depth analysis of changes in fertility before and after knowing about HIV status.

A significant proportion of women became aware of their HIV status soon after the death of their husbands (Cluster: HIV diagnosis after husband’s death) especially women who were married at younger age, had no or primary level education, were residing in rural area and were tested HIV positive prior to 2005. ART was made available in public health facilities since 2004 and it appears that the likelihood of women knowing about their HIV status soon after the death of their husbands has declined since then, probably because of the availability of treatment to the husband. However, the finding highlights the need and the challenges of early HIV testing among male partners and linking them to care facilities especially when the ART program is now being scaled up in rural areas among populations with relatively low education.

HIV infected young widows remain a significant, yet neglected population within HIV programs. A recent study (2014) from sub-Saharan Africa analyzed the relationship of marital status and risk of HIV acquisition among women and observed higher risk of HIV transmission among divorced women and widows [38]. Higher rates of HIV prevalence were also observed among widows in India in the nationally representative survey [25]. There is lack of research on the vulnerabilities of HIV infected Indian women who experience marriage dissolution either due to divorce or due to husband’s death. Previous research from Africa suggests higher risk of marriage dissolution due to divorce or separation among HIV infected women compared to uninfected women [39]. However, this pattern appears unlikely in India. Nonetheless, with increase in longevity of HIV infected women in India, it is critical to understand needs of widowed women including their sexual and reproductive needs in order to devise specific intervention.

A significant proportion of women in this study appears to have known their HIV positive status after completing their reproductive career, especially women who got married at a younger age and who had no or primary level education (Cluster: HIV diagnosis after childbearing). Widespread use of female sterilization at a young age could result in shorter effective reproductive span and can lead to such a trajectory of reproductive career among Indian women. With respect to HIV transmission, negotiating condom use for preventing sexually transmitted infections (STIs) could be challenging when there is no apparent risk of pregnancy. Therefore, there is a need to devise strategies and programs for HIV testing of men and women who have completed their reproductive career (hence who would not access routine ANC testing in the PMTCT program). This pattern of getting diagnosed after completing childbearing, which did not significantly change over a period of time, also has implications for assessing the representation of HIV infected women in ANC which is the main source of estimating HIV prevalence in the country since the beginning of HIV epidemic [16]. With this pattern it can be expected that there would be a significant number of women acquiring HIV after completing their reproductive career and would not be represented in ANC. On the other hand extrapolating HIV prevalence estimated among pregnant women attending ANC to the general population might underestimate HIV prevalence in the latter group.

#### Cumulative disadvantage

The probability that HIV infected women will follow a specific trajectory within their reproductive career seems to be associated with factors that generally put women into disadvantaged position with respect to health. Factors such as lower education and rural residence that were conclusively associated with health care utilization among Indian women also seem to be significant factors determining their access to HIV testing services and subsequent interaction with the events in the reproductive career. Though these factors have been identified in most previous studies on utilization of maternal and child health care services among HIV infected and uninfected women [35,40], little is known about the mechanisms through which these factors, particularly lower education, influences women’s decision-making process related to care seeking. Along with further research to identify pathways in which education may influence health care utilization, it is programmatically essential to identify strategies that can increase uptake of services among women with lower education. The role of HIV related factors such as stigmatization and discrimination in affecting care seeking should be investigated further [41].

### Limitations

Similar to most clinic based studies of HIV, this study has a selection bias as women who are diagnosed early and linked to care are likely to be over-represented, leaving out women who could never reach the clinic. Our sample of ever married women who knew about their HIV status for more than 6 months also induces bias due to censored observation among women who remained unmarried and events among women who remained undiagnosed (for example HIV uninfected women with HIV infected partner). Also, the trajectories of all individuals included in the study are not fully completed (due to different ages at the time of the interview). Yet the inclusion of the observable exposure time of these individuals remains informative and provides important insights about the timing and sequencing of reproductive events in the life of HIV infected Indian women.

While reporting the reproductive events, women had to recall the timings of these events from the period of their marriage till the date of interview. The average recall period was 15 years. However, the adoption of conversational interview techniques and the retrospective calendar method, which have been shown to reduce recall bias [42] due to the flexibility of the method to collect temporal information on life events and its structural alignment with the respondent’s recall process, likely helped in collecting more reliable information. Also, information regarding partner’s HIV status was collected from women, which can be considered not ideal. However, the study was conceptualized from the women’s perspective and all the other information regarding reproductive behavior was collected from the women. Hence women’s knowledge about partner’s HIV status was considered more important than the actual status.

## Conclusion

The diachronic and longitudinal approach of the analysis of interaction of HIV diagnosis with the childbearing trajectory is fruitful in describing the complexity in timing and sequencing of reproductive events in the life of HIV infected women and is helpful in identifying typical patterns in the trajectories. The distinct pattern of timing of reproductive events and HIV transmission among women make the results of this study specific to India. HIV and childbearing are closely timed events only for a group of women who get HIV testing during pregnancy and are generally represented in the PMTCT programs. There appears to be a significant group of women who become aware of their HIV positive status after they complete childbearing or even at the time of death of their husband. This suggests the need to increase opportunities for men and women to get tested for HIV early in their reproductive career. Also, HIV testing and prevention programs should target a group of men and women who acquire HIV infection after childbearing as these women are not included in the PMTCT program. Due to widespread use of female sterilization at an early age, negotiating condom use for prevention of STIs could be challenging for this group.

## Supporting Information

S1 Data Background data of HIV infected women enrolled in the study (N = 622). (XLSX)

S2 Data State sequence data of HIV infected women from birth till date of interview (N = 622). (CSV)
